# Extracellular Vesicles and Thrombogenicity in Atrial Fibrillation

**DOI:** 10.3390/ijms23031774

**Published:** 2022-02-04

**Authors:** Alexander E. Berezin, Alexander A. Berezin

**Affiliations:** 1 Internal Medicine Department, State Medical University, Zaporozhye 69035, Ukraine; 2 Internal Medicine Department, Medical Academy of Postgraduate Education, Zaporozhye 69096, Ukraine; lunik.mender@gmail.com

**Keywords:** atrial fibrillation, coagulation, thromboembolic complications, extracellular vesicles

## Abstract

Extracellular vesicles (EVs) are defined as a heterogenic group of lipid bilayer vesicular structures with a size in the range of 30–4000 nm that are released by all types of cultured cells. EVs derived from platelets, mononuclears, endothelial cells, and adipose tissue cells significantly increase in several cardiovascular diseases, including in atrial fibrillation (AF). EVs are engaged in cell-to-cell cooperation, endothelium integrity, inflammation, and immune response and are a cargo for several active molecules, such as regulatory peptides, receptors, growth factors, hormones, and lipids. Being transductors of the intercellular communication, EVs regulate angiogenesis, neovascularization, coagulation, and maintain tissue reparation. There is a large amount of evidence regarding the fact that AF is associated with elevated levels of EVs derived from platelets and mononuclears and a decreased number of EVs produced by endothelial cells. Moreover, some invasive procedures that are generally performed for the treatment of AF, i.e., pulmonary vein isolation, were found to be triggers for elevated levels of platelet and mononuclear EVs and, in turn, mediated the transient activation of the coagulation cascade. The review depicts the role of EVs in thrombogenicity in connection with a risk of thromboembolic complications, including ischemic stroke and systemic thromboembolism, in patients with various forms of AF.

## 1. Introduction

Atrial fibrillation (AF) is the most common form of cardiac arrhythmia amongst older people and patients with cardiovascular (CV) diseases (CVD) and continues to demonstrate steady growth in the general population [1]. The prevalence of AF is increasing at epidemic proportions in both developed and developing countries, regardless of the presence of conventional CV risk factors [2,3]. Indeed, the current prevalence of AF in the European Union is 7.3%, and it will increase to 89% by 2060 [1,3]. Moreover, at least 65% of senior citizens in the European Union will have AF in 2060, and paroxysmal, persistent, and permanent forms of AF are projected to be diagnosed in approximately 5,989,000, approximately 2,833,000, and approximately 5,579,000 of older people, respectively [3]. Electroanatomic and adverse cardiac remodeling resulting in natural CVD evolution and AF persistence is considered to be a substrate for the development of cardiac dysfunction, heart failure (HF) occurrence, and thromboembolic complications, which sufficiently reduce life span duration and quality of life in patients affected by this condition [4]. In addition, AF is independently associated with both high rates of morbidity, and mortality is a common cause of premature disability in CVD patients [5]. Overall, the risk of thromboembolism in these patients is not just associated with AF, but it also varies widely depending on coexisting comorbidities (HF, chronic kidney disease, chronic obstructive pulmonary disease, diabetes mellitus, obesity, and cardiomyopathy), low physical activity, patient age and gender, and methods of cardioversion and anticoagulation [6,7,8]. Despite the numerous benefits of CVD prevention therapy and its wide implementation in routine practice, there remains an unacceptably high risk of potentially devastating complications associated with catheter pulmonary vein isolation (PVI), such as stroke/transient ischemic attack and systemic thromboembolism during persistent AF or the occurrence of its permanent form [9]. The number of one-year recurrent AF episodes has remained high (20%) [10], whereas single-procedural 1-year and 5-year arrhythmia-free survival is 66% and 44%, respectively [11,12]. However, the prevention of thromboembolic complications remains the focus of pragmatic strategy development for AF therapy [13,14].

The extracellular vesicles (EVs) are vesicular structures that are secreted from numerous cells and supply several biologically active molecules (growth factors, active peptides, regulatory proteins, pro-inflammatory cytokines, micro-RNAs (miR)) that are involved in cell-to-cell communication, including in the modulation of tissue repair, inflammation, angiogenesis/neovascularization, immune response, extracellular matrix accumulation, and vascular integrity enhancement [15,16,17]. Moreover, EVs that originate from the activated or apoptotic cells exert variable effects that are dependent on a spectrum of encapsulated cytokines, proteome, lipidome, and miRs and have an epigenetic impact on target cells [16]. There is strong evidence of the fact that EVs that are derived from a large spectrum of circulating blood cells, including platelets, mononuclear cells/leucocytes, endothelial cells, and even adipose tissue cells and antigen-presenting cells, are a cargo for pro-coagulant phospholipids, mainly phosphatidylserine, active molecules, non-coding RNAs, and other components, that regulate coagulation cascade and play a crucial role in the incidence of AF-related thromboembolism [15,16,17]. This review recapitulates recent findings on the role of EVs in thrombogenicity in connection with a risk of thromboembolic complications, including ischemic stroke and systemic thromboembolism, in patients with various forms of AF.

## 2. Methodology

We searched the MEDLINE, EMBASE, Medline (PubMed), Web of Science, and Cochrane Central bibliographic databases with the keywords [exosome AND atrial fibrillation], [extracellular vesicles AND atrial fibrillation], [extracellular vesicles AND thrombogenicity] and found a total of 12 original articles written in English. We evaluated the quality of each article to ensure that the data were homogenous. We used the following criteria to determine the quality of eligible papers: full-length manuscript with an English abstract and published in a reputable journal, human study, data that were available for assessment, the presence of a control group, a clear and concise description of the methods by which EVs were determined and measured, and a lack of result misinterpretation. In the present study, we focus on original articles addressing EVs and AF.

## 3. Extracellular Vesicles: Definition, Nomenclature, Biological Function

According to the International Society on Extracellular Vesicles (ISEV), EVs are defined as a heterogenic group of lipid bilayer vesicular structures with a size in the range of 30–4000 nm that are released by all types of cultured cells and are found in abundance in body fluids (blood, lymph, saliva, urine, bile, synovial fluid, cerebrospinal fluid) [18]. EVs consist of three subpopulations: exosomes, microvesicles (MVs), and apoptotic bodies (ABs), that can be distinguished from each other based on their size, immune phenotypes, origin, biogenesis, and mechanism of release and component delivery [19,20,21]. Table 1 contains the main characteristics of EVs. Although a universal definition of EVs subpopulations remain elusive, the ISEV recommends not using other criteria apart from the size of the EVs to classify them because there is overlap among the different phenotypes of these vesicular structures [22]. Biological pathways of generation, shedding, and the release of EVs exert remarkable diversity. For instance, exosomes appear following endocytosis from endosomes, whereas MVs are synthesized after blebbing from the plasma membrane and being released in biological fluid and contain all of the antigens that are widely expressed on the surface of the mother cells [23]. In addition, MVs and exosomes are not only produced by cellular activation, but they are also produced via pro-inflammatory stimulation, shear stress, and the influence of pro-thrombotic and pro-apoptotic substances. ABs occur as the result of the shrinkage and blebbing of apoptotic cells, but there is alternative pathway that relates to lysosome vesicle secretion and secretory autophagy. Therefore, mechanical activation and hemolysis, which are common features in patients with CVD (valve stenosis, prosthetic valves, AF, HF) as well as non-CVD (infections, sepsis, and eclampsia), are strongly associated with apoptotic cell breakdown [24,25,26,27]. In fact, an evaluation of the EVs secretome demonstrated that different sub-populations of EVs had either overlapping protein components or coincidentally similar protein arrangements, and this was a consequence of the type of stimulation that caused the EVs to release and the type of the mother cells [28].

The exact circulation levels of the different EVs subpopulations in healthy individuals and in patients with CVD and non-CVD is unclear due to a lack of strong evidence regarding their concentrations due to high variability in the sensitivity and specificity of the detection methods used or the co-detection of contaminants [29]. However, it is widely agreed upon that the majority of circulating EVs are of a platelet-derived vesicular structure, whereas neutrophil-derived, mononuclear-derived, endothelial cell-derived, and red blood cell-derived vesicles have been found in by far lower concentrations when compared to platelet-derived ones [30].

**Figure 1:**
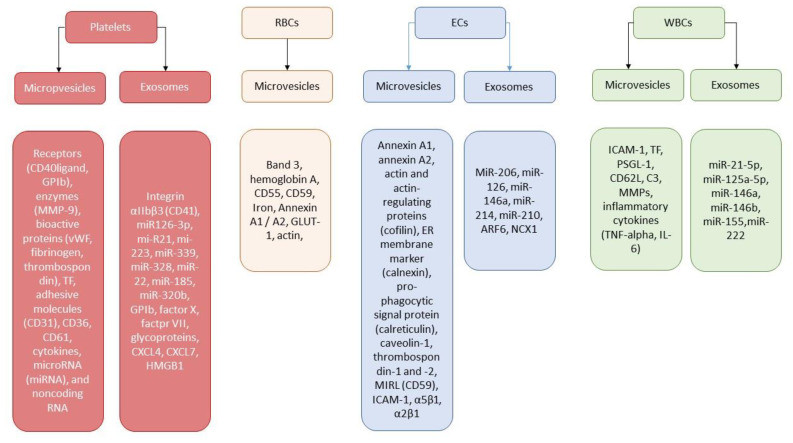
Secretome of EVs originated from different cells. Abbreviations: ARF6, ADP ribosylation factor 6; ECs, endothelial cells; RBCs, red blood cells; ER, endoplasmic reticulum; ICAM-1, intracellular adhesion molecule 1; CD62L, l-selectin; MIRL, membrane inhibitor of reactive lysis; MMPs, metalloproteinases; TNF-alpha, tumor necrosis factor-alpha; TF, tissue factor; vWF; Von Willebrand factor; CXCL, C-X-C motif ligand; HMGB1, high mobility group box 1; PSGL-1, P-selectin glycoprotein ligand-1; WBCs, white blood cells.

Nowadays, EVs are considered to be powerful mediator of cell-to-cell communication through the delivery of cargo proteins (adenosine diphosphate-ribosylation factor 6, Ras-related protein 22a, vesicle-soluble NSF attachment protein receptor, vesicle-associated membrane protein 3, T-cell internal antigen 1, argonaute-2, lipids, mRNA, miRNA long noncoding RNA, and occasionally genomic DNA) [31]. In addition, EVs directly transfer functional receptors, such as CD41, CD61, CD62, CXCR4, PAR-1, and PPRγ, from parental cells to recipient cells, contributing an axis for the regulation of their proliferation, differentiation, and activation. A large spectrum of miRNAs was observed in the EVs that were isolated from human parental cells. More often than not, the miRNAs that are incorporated into EVs act as negative regulators for several biological processes, including tissue reparation, vascular integrity, angiogenesis, neovascularization, immune response, and inflammation. Indeed, miR126-3p is responsible for the suppression of the expression of different inflammatory genes in the macrophages/mononuclears and thereby mediates cytokine-induced thrombogenicity and NETosis [32]. Platelet-derived EVs enriched in miR 21, miR-223, and miR-339 seem to be a modulator of expression of the platelet-derived growth factor receptor-beta in smooth muscle cells, playing a crucial role in plaque development and vascular remodeling [33]. Figure 1 illustrates the role of EVs’ secretome depending on the origin of the vesicles.

## 4. Extracellular Vesicles and Thrombogenicity/Thrombosis

**Figure 2:**
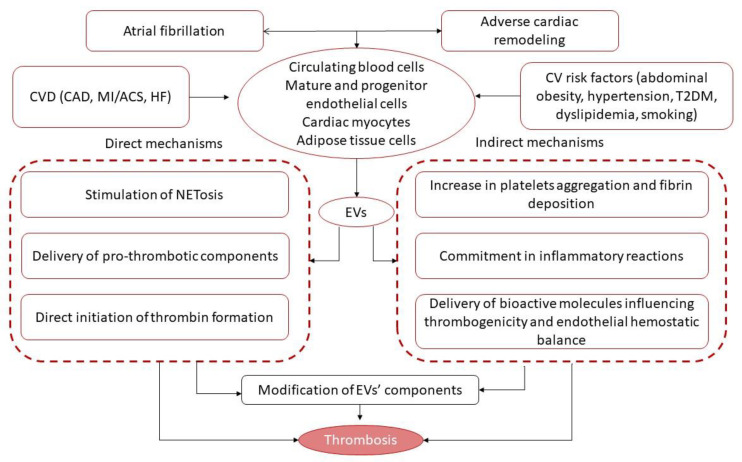
Pathogenetic pathways underlying EV-related thrombosis formation and increase in thrombogenicity. Abbreviations: CAD, coronary artery disease; ACS, acute coronary syndrome; EVs, extracellular vesicles; NETosis, neutrophil extracellular traps; AF, atrial fibrillation; CVD, cardiovascular diseases; CV, cardiovascular; HF, heart failure; T2DM, type 2 diabetes mellitus.

It has been suggested that cell-derived EVs that are enriched in lipids (phosphatidylserine, arachidonic acids), secretory phospholipase A2, factors of coagulation (factor X, factor VII, tissue factor), chromatin, and DNAs/RNAs might have 50- to 100-fold higher specific pro-coagulant activity than activated platelets [30,34]. The mechanisms by which these EVs exert their pro-coagulative abilities are considered to be quite complex (Figure 2).

First, secreted EVs may facilitate the formation of neutrophil extracellular traps (NETs), which may contribute to thrombus development and attenuate the counteracting effect of endogenous fibrinolytic systems [35,36]. Second, the presence of polyphosphate (polyP) in cell-derived EVs may promote thrombosis through a tissue factor-independent route, whereas these effects were initially described in cancer-associated thrombosis [37] and then extrapolated to others. Yet, the pro-coagulant activity to EVs that originated from mature endothelial cells was found to be up-regulated by the induction of the expression of adhesion proteins and several encapsulated pro-inflammatory cytokines, mainly IL-8 and tumor necrosis factor-alpha (TNF-alpha), on mother cells [38]. These mechanisms are rigorously regulated by the protease-activated receptor (PAR) 2 signaling pathway, which, in turn, mediates the rapid generation of pro-thrombotic components, such as inactive tissue factor/factor VII and integrin α5 β1 into the EVs secreted by endothelial cells [39]. Finally, the internationalization of the pro-thrombotic complex consists of tissue factor–factor VIIa–factor Xa by endothelial cells and regulates tissue factor availability for release on pro-coagulant EVs [40,41]. Third, the P2Y1 and P2Y12 receptors, which play a pivotal role in platelet activation and aggregation, seem to be transported by EVs as membrane-associated structures [42]. The next thrombogenicity-stimulating pathway is direct thrombin activation by EVs enriched by chemokines (CXCL4, CXCL7) and the cytoplasmic high-mobility group box 1 protein [43]. In addition, platelet-derived and endothelial cell-derived MVs and exosomes can stimulate coagulation independently after the mechanical stimulation of the parental cells or due to hemolysis [44].

Therefore, EVs can potentiate thrombosis through several indirect mechanisms, such as through an increase in platelet aggregation and fibrin deposition following the transfer of arachidonic acid [45], the down-regulation of endothelial cyclooxygenase (COX)-2 expression and prostacyclin synthesis via thromboxane A2-related mechanisms [46], and increased endothelial cell surface thrombogenicity and altered endothelial hemostatic balance [47]. It has been found that platelet-derived MVs are able to modulate the expression of (COX)-2 and prostacyclin production in both circulating monocytes and in progenitor/mature endothelial cells through the direct activation of the PKC/p42/p44 MAPK/p38 kinase and c-Jun N-terminal kinase/Elk-1pathways [48]. Indeed, these pathways contribute to vascular homeostasis through the regulation of plasmin generation on the endothelial cell layer and is a crucial player in ensuring thrombogenicity and blood clotting control [49]. The EVs that are mainly derived from endothelial cells participate in transfer growth factors (fibroblast growth factor, transforming growth factor-beta), active proteins (including annexin A2 ligand and CD40 ligand+), and receptors, resulting in TNF-alpha and IL-6 co-stimulation, and, in turn, lead to plasmin generation and the expression of both the urokinase-type plasminogen activator (uPA) and its receptor (uPAR) on the surface of endothelial cells [50]. All of these trigger an increase in their ability to bind exogenous uPA on uPAR and to subsequently maintain plasmin formation. Moreover, this was found to be a signal messenger for endothelial cell-derived EVs to induce plasmin generation and, in turn, affect tube formation in endothelial progenitor cells, thereby ensuring their proliferative and proteolytic activities [49]. Previous studies have shown that S100A10 is a member of the S100 family of Ca^2+^-binding proteins and was found to be abundantly distributed in progenitor and mature endothelial cells and protected from altered vascular integrity and hypercoagulation [51,52]. Indeed, this resulted in the loss of S100A10 from the endothelial cells, which exerted significant plasmin generation suppression [51,52,53]. It is possible that the EVs originating from activated endothelial cells are able to be a cargo for the kringle-2 domain of tPA, which plays a crucial role in S100A10-dependent plasmin generation [54]. Thus, decreased number and lowered functional activity of endothelial cell-derived EVs are considered to be a powerful factors for increased thrombogenicity.

These mechanisms based on both the synthesis and transfer of coagulation factors by EVs, but these can also be activated via the pro-inflammatory cytokine cascade, the proteasome-dependent mechanism, and apoptosis [55]. In fact, It is prudently suggested that these mechanisms overlap with each other. For instance, pro-inflammatory cytokine IL-33 and cholesterol were found to be powerful inductors for differential tissue factor expression and stimulators for monocyte subsets as well as for the release of pro-coagulant EVs into circulation from mother cells [55,56]. Consequently, inflammatory cytokines along with other factors generated during blood coagulation, such as platelet-derived lipid mediators (lysophosphatidate, phosphatidic acid and sphingosine 1-phosphate), may contribute to the formation of a pro-thrombotic state in CVD patients as well as potentiate neovascularization and angiogenesis [57]. Considerable attention has focused on the ability of pro-coagulant lipid components, mainly phospholipids derived through EVs, to be agonists for G-protein-coupled endothelial differentiation gene receptors, which seem to be important for the morphogenesis of capillary-like structures, promoting angiogenesis and supporting blood clotting [57]. On the other hand, numerous EVs enriched in phospholipids have been derived from activated platelets, resulting in blood coagulation and were eventually noticed to be key modulators of the chemo-attractive and proteolytic activity of the endothelial cells [58,59] that linked CV factors, comorbidities, endothelial integrity, and thrombogenicity [60].

Another pathophysiologic mechanism that would explain the link between AF and thrombogenicity is the indirect influence of the EVs on thromboembolic complications through adverse cardiac remodeling. Indeed, there is a wide range of resoundingly clear scientific proof of the fact that EVs enriched in cardiac protective microRNAs, such as miR-30d, miR-17-3p, miR-155, miR-222, and miR-378, are primary secreted by cardiac myocytes following acute-phase ischemia, hypoxia, inflammation, and biochemical stress and may ameliorate apoptosis through the activation of MAP4K4 (mitogen-associate protein kinase 4), the down-regulation of tumor protein p53-inducible nuclear protein 1, and the suppression of cardiac fibroblast proliferation and activation by directly targeting integrin α5 [61,62,63]. On the contrary, the chronic phases of these conditions were strongly associated with lower miR-30d expression in the myocardium and also decreased the amount of circulating EVs enriched in miR-30d, miR-17-3p, and miR-222, something that that is considered to be related to adverse cardiac remodeling in animal models and humans [62,63,64]. All of these are consequently linked to the over-expression of the genes that are implicated in fibrosis and inflammation and might play a role in the occurrence of non-valvular AF [65]. In addition, lowered levels of circulating endothelial cell-derived EVs, mainly the MVs that are able to regulate myocardial reparation and vascular integrity through supplying active molecules, regulatory peptides, miRNAs, and growth factors, were found to be predictors of adverse cardiac remodeling and AF [66,67]. In addition, the EVs derived from adipose tissues, including epicardial fat, as well as those originating from apoptotic mononuclear cells, were found to have encapsulated certain inflammatory cytokines, such as TNF-alpha, IL-1α, IL-1β, and RANTES, and they were also determined to have a cytotoxic impact on the myocardium, promoting left ventricular hypertrophy and arhythmogenesis [68,69]. Thus, the EVs originating from cardiac myocytes and circulating blood, mainly endothelial cells and mononuclear cells, may indirectly influence the development of AF through the mediation of cardiac hypertrophy, fibrosis, and oxidative stress/inflammation and aggravating atrial remodeling.

## 5. Atrial Fibrillation and Signature of EVs

Atrial fibrillation seems to be a powerful trigger for a pro-thrombotic state, which is associated with shedding EVs from many activated and apoptotic cell types in response to blood turbulence, inflammation, hemolysis, and adverse cardiac remodeling as well as an effect on coexisting CV risk factors and diseases, including HF, stroke, and atherosclerosis [70,71,72,73,74]. The changes in the EV signature in patients with different forms of AF are reported in Table 2.

### 5.1. Signature of EVs in Non-Valvular AF

Shaihov-Teper, O. et al. (2021) [69] investigated the signature of epicardial adipose tissue-derived EVs in patients with AF and found a significant difference between AF patients and individuals without AF in terms of the compounds of pro-inflammatory and pro-fibrotic cytokines and pro-fibrotic miRNAs encapsulated in the EVs. The authors concluded that the EVs from AF patients had more a profound pro-inflammatory, pro-fibrotic, and pro-arrhythmic epicardial adipose tissue-derived-EVs signature than patients without AF. [69]. Siwaponanan, P. et al. (2019) [75] reported that patients with non-valvular AF had sufficiently higher levels of total circulating MVs, platelet-derived MVs, and endothelial cell-derived MPs compared to healthy volunteers, even after adjusting for potential co-factors. The authors also noticed that the levels of circulating MVs enriched in pro-coagulant lipids (phosphatidylserine) did not significantly differ between the AF patients and healthy volunteers. On the contrary, there is a large amount of evidence regarding the fact that circulating pro-coagulant MVs were found in abundance in patients with persistent and/or permanent AF who were not receiving anticoagulant therapy compared to age-matched control subjects [76,77,78]. It is interesting to note that a number of platelet-derived MVs and endothelial cell-derived MVs were not distinguished in patients with non-valvular AF and control subjects with CV risk factors, but the total amount of EVs was significantly higher in the AF patients than it was in in healthy volunteers without known CV risk factors [76,77,78]. However, the presence of AF was found to be a solid predictor of the annexin V(+) MVs level, which seems to be a marker for hypercoagulation and a risk of both atrial thrombosis and systemic thromboembolism. Therefore, there is strong evidence for the fact that the amount of platelet-derived MVs was positively correlated with the thrombus diameter in non-valvular AF patients [79]. In another study, Wang, H. et al. (2020) [80] established that the levels of platelet-derived MVs in high-risk stroke patient according to the CHADS2 score were significantly higher compared to low to moderate risk AF patients. Finally, the data of the patients who were included in the Pulmonary Embolism Response Team (PERT) registry revealed that EVs may be a promising predictive indicator for acute pulmonary thromboembolism episodes in non-valvular patients with AF [88], but the clinical significance of the findings need elucidation in large-scale clinical trials.

### 5.2. Changes in EVs Profile during Anticoagulation Therapy in AF Patients

Lenart-Migdalska, A. et al. (2021) [81] investigated the impact of rivaroxaban on the circulating levels of both platelet-derived MVs (CD42b) and endothelial cell-derived MVs (CD144) amongst patients with non-valvular paroxysmal, persistent, or permanent AF (CHA2DS2-VASc score ≥ 2).

The authors found that the chronic administration of rivaroxaban was associated with an increase in the circulating number of both MVs phenotypes and that the peak plasma concentration of rivaroxaban was well correlated to the amount of MVs. In confirmation of these findings, Lenart-Migdalska, A. et al. (2020) [81] reported that dabigatran administration was associated with sufficient increases in the circulating levels of platelet-derived MVs (CD42b) in AF patients, whereas the amount of endothelial cell-derived (CD144) MVs was not elevated. The results of the West Birmingham Atrial Fibrillation Project yielded that the AF patient group had increased levels of apoptotic MVs compared to the disease control group and that warfarin and apixaban were similar in terms of their effect of reducing the pro-thrombotic index [82]. In fact, these findings clarify a possible pro-thrombotic role of MVs in oral anticoagulants, such as rivaroxaban, apixaban, and dabigatran, and that requires further investigation in large-scale clinical studies. The study by Duarte, R. et al. (2021) [83] revealed that patients with non-valvular AF undergoing warfarin or rivaroxaban treatment had higher levels of platelet-derived MVs; however, there was no difference in the levels of endothelial cell-derived MVs between the groups compared to the age- and sex-matched controls. These data may be important to stratify AF patients who are at risk of thromboembolic complications during anticoagulation therapy.

### 5.3. EV Signature in Patients with Valvular AF

There are limited data describing the changes that take place in the EV signature in valvular AF. Azzam H. and Zagloul M (2009) [84] reported that the levels of circulating platelet-derived MVs, labeled as CD41(+) particles, were significantly elevated in patients with rheumatic mitral stenosis and any form of AF compared to healthy volunteers. In addition, the authors noticed a significant correlation between the severity of mitral stenosis and circulating levels of platelet-derived MVs.

### 5.4. The Levels and Immune Phenotypes of EVs in AF Patients Treated with Catheter Ablation

However, it remained unclear whether patients with AF had an increased thrombotic status depending on the right and left atrium volume after pulmonary vein isolation. Jesel, L. et al. (2014) [85] no found atrial-specific differences in terms of the total levels of circulating pro-coagulant MVs, leukocyte-derived-MVs, or platelet-derived MVs. However, the authors noticed that the total amount of endothelial cell-derived MVs along with tissue factor activity and collagen-induced platelet aggregation were slightly increased in the right atrium and not in the left atrium. These findings are confusing to thoroughly explain the role of circulating pro-coagulant MVs in cardiac thrombus formation in non-valvular AF after pulmonary vein isolation. In addition, MVs-associated tissue factor activity was found to be reduced, whereas the fibrinolytic activity of the MVs in patients with acute episodes of AF treated with catheter ablation was improved [86]. On the contrary, Zhang, X. Z. et al. (2018) [87] reported that the hypercoagulable state after radiofrequency catheter ablation was significantly increased and exhibited a positive correlation with the total number of platelet-derived CD62P MVs. Moreover, it was revealed that the levels of platelet-derived MVs (CD62P) seven days after radiofrequency catheter ablation were significantly higher compared with those patients who were examined immediately after the procedure.

## 6. EVs in Routine Practice: Potential Benefits and Pitfalls

The findings support an emerging paradigm that involves EVs in thrombotic complications in patients with different forms of AF. Despite the fact that thrombogenicity can be directly modulated by platelet-derived EVs, resulting in altered hemodynamics, hemolysis, and blood turbulence as well as AF occurrence, an impaired circulating EV profile was found to be a promising biomarker for adverse cardiac remodeling, including left ventricular hypertrophy and fibrosis as well as a higher risk of AF [67]. Moreover, serum-derived EVs have been thoroughly identified as carrying potential biological markers, such as microRNAs, for the diagnosis and treatment of AF, especially with oral anticoagulants, but their clinical significance requires further investigations to clearly understand the reproducibility, sensitivity, and specificity of the targets [89]. Although specific markers and the methodology for determining various subsets of EVs have also been thoroughly described [22], there are no clear recommendations for a routine laboratory practice to measure EVs. In addition, there is no consent between investigations as to how to interpret and compare the results and whether a determination of an altered signature of EVs is better than single subset of ones to predict CV events in AF patients with and without HF. Indeed, some comorbidities that were found to have been frequently associated with AF, such as type 2 diabetes mellitus, abdominal obesity, and HF, exhibited an altered EVs profile along with impaired EVs proteinomics and lipidomics. Whether the secretome of different cells, including EVs, exerts a unique perspective for the diagnosis of a pro-thrombotic state in AF patients, regardless of comorbidities, remains uncertain. Another unresolved issue is the economic burden that occurs when a new biomarker is implemented into clinical practice. Unfortunately, there is no strong evidence to support that fact that the EVs signature is a superior conventional biomarker to predict thromboembolic complications, such as cardiac thrombosis, stroke, and systemic thromboembolism. All of these need to be investigated in large-scale clinical studies in the future.

## 7. Conclusions

Circulating EVs that are enriched in pro-coagulant components appear to be powerful triggers of cardiac thrombus formation and systemic thromboembolism. The altered signature of circulating EVs that are mainly derived from apoptotic cells and activated platelets and endothelial cells was found to be a promising predictor of potential thromboembolic complications, although the impact of different anticoagulants on the dynamic of these biomarkers is uncertain and requires clear elucidation in large clinical trials. The wide implementation of catheter ablation may require more advanced methods for the re-evaluation of potential thromboembolic risk, and in this context, EVs monitoring seems to be remarkably effective, but this approach needs validation in further investigations.

## Figures and Tables

**Table 1:** Characteristics of EVs.

Characteristics	Exosomes	Microvesicles	Apoptotic Bodies
Diameter, nm	30–150	100–1000	500–4000
Sedimentation, g	100,000	20,000	16,000
Pathway for biogenesis	Endocytosis from endosomes and exocytosis of late endosomes/MVBs	Blebbing from plasma membranes	Shrinkage and blebbing of apoptotic cells
Unconventional secretion pathway	Cellular activation	Early apoptosis	Lysosome vesicle secretion and secretory autophagy
Delivery contents	Alix, chaperones, Rab proteins, Rab GTPases, SNAREs, lipid rafts, proteins (flotillin), myokines, inflammatory cytokines, growth factors, miRs.	Arachidonic acid, cytokines, chemokine RANTES/CCL5, P-selectin, lipids, signaling proteins, miRNA, and microRNA, membrane-anchored receptors (PPARγ) and adhesion molecules	Organelles and/or nuclear content including chromatin, DNA, miRNAs, microRNAs, histones, oncogenes.
Membrane-specific antigens	Tetraspanins (CD9, CD81, CD63), TSG101,	Integrins, selectins, membrane proteins of parental cells	Annexin-V(+)

Abbreviations: Alix, ALG2 interacting protein X; TSG101, tumor susceptibility gene 101; SNAREs, soluble N-ethylmaleimide-sensitive factor attachment protein receptors; PPARγ, peroxisome proliferator-activated receptor γ; MVBs, multivesicular bodies; RANTES, Regulated upon Activation, Normal T Cell-Expressed and Presumably Secreted; miRs, micro-RNAs; (+), positive.

**Table 2:** The signature of EVs in patients with non-valvular AF.

Type of AF	Study Design	*n*	Comparator(s)	Anticoagulation Therapy	Results	Reference
Paroxysmal, persistent, or permanent, non-valvular AF	Cohort study	32 AF patients	30 patients without AF	Non-treated	EVs received from AF patients had distinctive pro-inflammatory, pro-fibrotic, and pro-arrhythmic signature of epicardial adipose tissue-derived-EVs	[69]
Paroxysmal, persistent, or permanent, non-valvular	Cohort study	66 AF patients	33 healthy volunteers	Non-treated	↑ total circulating MVs, platelet-derived MVs, endothelial-derived MVs. No difference in lipid enriched MVs between AF patients and healthy volunteers	[75]
Permanent and/or persistent, non-valvular	case–control study	45 AF patients, 90 control individuals	45 with CV risk factors and 45 without	Non-treated	↑ Annexin V-positive MV levels	[76]
Paroxysmal, persistent, or permanent, non-valvular	Case-control study	70 AF patients	46 disease control subjects and 33 healthy control subjects	Non-treated	↑ number of platelet-derived MVs, but no difference between AF patients and disease control subjects	[77]
Paroxysmal, persistent, or permanent, non-valvular	Cohort study	37 AF patients (11 and 23 patients treated with 15 mg and 20 mg of rivaroxaban)	11 patients (15 mg of rivaroxaban)	Rivaroxaban 15–20 mg daily	↑ CD144(+) and CD42b(+) MV levels	[78]
Paroxysmal, persistent, or permanent, non-valvular	Cohort study	78 AF patients	36 controls	18 AF patients with thrombi versus 60 AF patients without ones	The amount of platelet-derived MVs was positively correlated with thrombus diameter	[79]
Paroxysmal, persistent, or permanent, valvular	Cohort study	210 AF patients	‘low to moderate risk’ compared to ‘high risk’ for stroke according to the CHADS2 score	AVK/OAK	↑ levels of platelet-derived MVs in high-risk patients compared with low to moderate risk patients	[80]
Paroxysmal, persistent, or permanent, valvular	Cohort study	39 AF patients with CHA2DS2-VASc score ≥ 2	11 patients with dabigatran of 110 mg bid versus 28 patients with dabigatran of 150 mg bid	Dabigatran for 3 months or more	↑circulating levels of platelet-derived MVs (CD42b) without changes in endothelial cell-derived (CD144) MVs	[81]
Paroxysmal, persistent, or permanent, valvular	Cohort study	120 AF patients naïve to oral anticoagulants	62 AF patients treated with antiplatelets	AVK/OAK/antiplatelets	Warfarin and apixaban demonstrated comparable positive effects on the levels of apoptotic MVs	[82]
Paroxysmal, persistent, or permanent, non-valvular	Cohort study	60 AF patients	Age and sex matched controls	Warfarin or rivaroxaban	No difference in levels of endothelial cell-derived MVs between the groups. Patients taking rivaroxaban and warfarin had significantly higher platelet-derived EVs levels compared to control group.	[83]
Paroxysmal, persistent, or permanent, valvular	Case control study	20 AF patients	10 healthy volunteers who were in sinus rhythm	Non-treated	↑ CD41(+) platelet-derived MVs	[84]
Paroxysmal/persistent, non-valvular	Case control study	22 AF patients undergoing pulmonary vein isolation	16 paroxysmal AF versus 6 persistent AF	AVK	No atrial-specific differences in the levels of several subsets of MVs in the left atrium volume, but not in the right atrial	[85]
Paroxysmal/persistent, non-valvular	Case control study	37 AF patients referred for AF catheter ablation	paroxysmal (*n* = 21) and persistent (*n* = 16) AF patients referred for AF catheter ablation	AVK	↓ pro-coagulant and ↑ fibrinolytic activity of MVs after catheter ablation	[86]
Paroxysmal/persistent, non-valvular	Case control study	60 AF patients after radiofrequency catheter ablation	20 healthy volunteers	AVK/OAK	↑ levels of platelet-derived MVs (CD62P) in seven days after radiofrequency catheter ablation compared with immediate after the procedure	[87]

Abbreviations: EVs, extracellular vesicles; AF, atrial fibrillation; CV, cardiovascular; ↑, increase; ↓, decrease; MVs, microvesicles; *n*, number of eligible patients; AVK, antagonists of vitamin K; OAK, oral anticoagulants.

## Data Availability

Not applicable.

## Abbreviations

AF	atrial fibrillation
Alix	ALG2 interacting protein X
CV	cardiovascular
CVD	cardiovascular disease
EVs	extracellular vesicles
MAP4K4	mitogen-associate protein kinase 4
MVBs	multivesicular bodies
MVs	microvesicles
HF	heart failure
HMGB1	cytoplasmic high-mobility group box 1 protein
PE	pulmonary embolism
PPARγ	peroxisome proliferator-activated receptor γ
PVI	pulmonary vein isolation
RANTES	Regulated upon Activation, Normal T Cell-Expressed and Presumably Secreted.
SNAREs	soluble N-ethylmaleimide-sensitive factor attachment protein receptors
TSG101	tumor susceptibility gene 101
TNF	tumor necrosis factor
